# 7000-year-old evidence of fruit tree cultivation in the Jordan Valley, Israel

**DOI:** 10.1038/s41598-022-10743-6

**Published:** 2022-05-06

**Authors:** Dafna Langgut, Yosef Garfinkel

**Affiliations:** 1 Laboratory of Archaeobotany and Ancient Environments, Institute of Archaeology, and The Steinhardt Museum of Natural History, Tel Aviv University, 6997801 Tel Aviv, Israel; 2 Institute of Archaeology, The Hebrew University of Jerusalem, Mount Scopus, 9190501 Jerusalem, Israel

**Keywords:** Agroecology, Palaeoecology, Plant domestication

## Abstract

This study provides one of the earliest examples of fruit tree cultivation worldwide, demonstrating that olive (*Olea europaea*) and fig (*Ficus carica*) horticulture was practiced as early as 7000 years ago in the Central Jordan Valley, Israel. It is based on the anatomical identification of a charcoal assemblage recovered from the Chalcolithic (7200–6700 cal. BP) site of Tel Tsaf. Given the site’s location outside the wild olive’s natural habitat, the substantial presence of charred olive wood remains at the site constitutes a strong case for horticulture. Furthermore, the occurrence of young charred fig branches (most probably from pruning) may indicate that figs were cultivated too. One such branch was ^14^C dated, yielding an age of ca. 7000 cal. BP. We hypothesize that established horticulture contributed to more elaborate social contracts and institutions since olive oil, table olives, and dry figs were highly suitable for long-distance trade and taxation.

## Introduction

**Figure 1:**
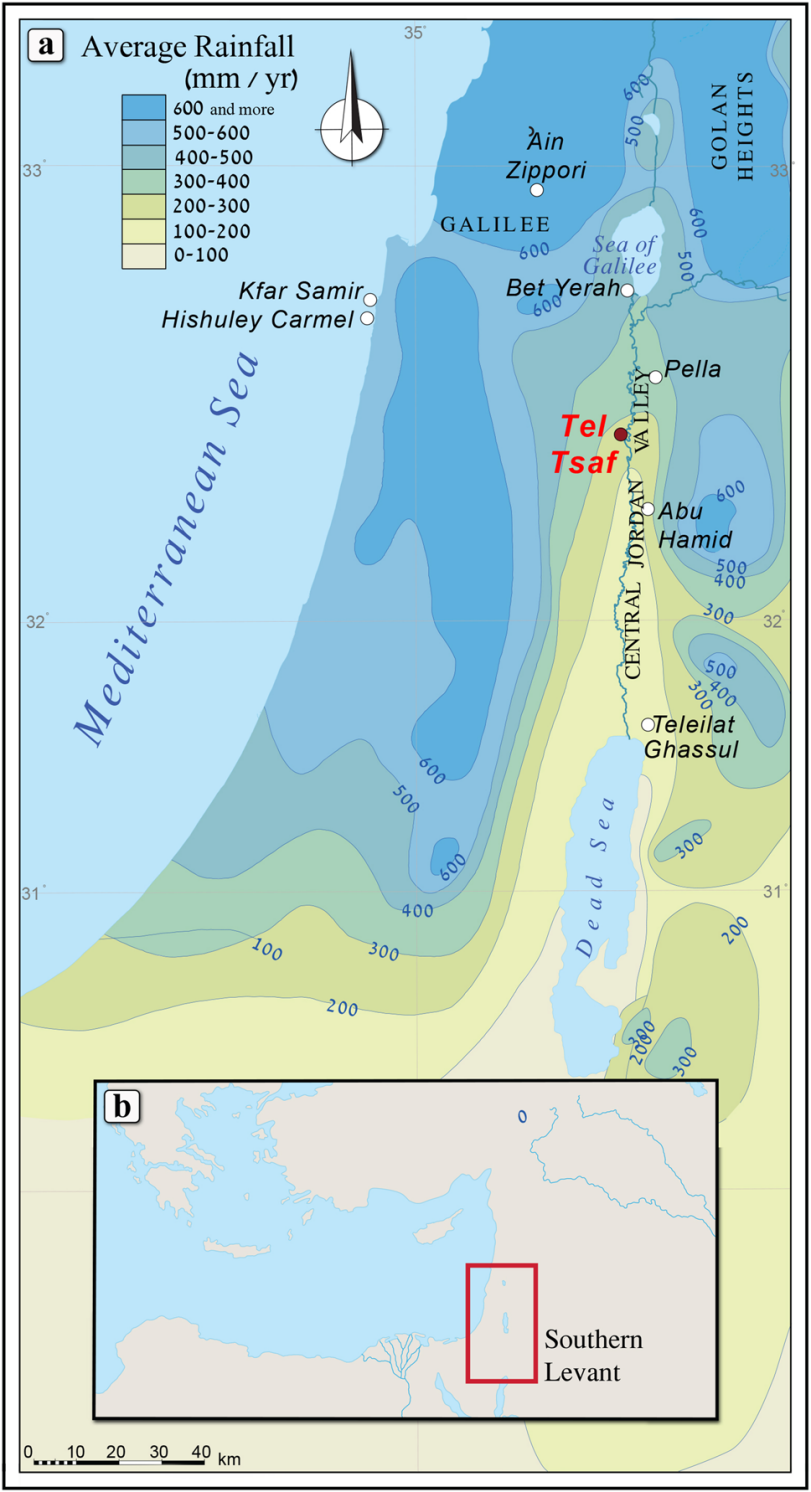
(**a**) Map of the southern Levant indicating mean annual precipitation in mm^25^; (**b**) the position of the southern Levant.

The late 8th/early 7th millennium BP site of Tel Tsaf, located at the Central Jordan Valley (Israel; Fig. 1), is significant not only because of its large size but also because of the presence of storage silos on a scale not previously unearthed in the Proto-historic Near East^1,2^. The material culture of the site is remarkably rich compared to contemporary sites in the region: dense concentrations of animal bones indicate large-scale feasts^3^; a unique and elaborate style of pottery decoration was common, consisting of red and black geometric designs on white slip^4^; a stone seal and some 140 seal impressions were found, including one vessel with two different seals^5,6^; two large concentrations of ostrich eggshell beads were found: ca. 900 in a courtyard and 1668 beads in a single grave^4^; some 100 stone beads were made of various green, red, and black minerals; additional imported substances and artifacts include raw greenstone chunks, Ubaid pottery from the northern Levant or Mesopotamia, obsidian from Anatolia, and Nilotic shells from Egypt^4^; a copper awl, the earliest in the Levant, was deposited as a grave good^7^.

The site’s splendid material culture and its participation in long-distance exchange were supported by the community’s economic organization, embodied by its extraordinary storage capacity. Each building had 4–5 rounded silos, amounting to 20–30 tons storage capacity. They greatly exceeded the inhabitants’ needs, indicating the operations of a complex economic system of surplus and wealth accumulation^1,2^. The location of the silos within individual courtyard buildings suggests a degree of coordination and management of the agricultural system at the site. Additional evidence of this comes from the seed assemblage. The flotation samples from the silos contained cultivars and larger wild cereals but almost no small weed seeds or cereal processing debris, meaning that the cereals must have been fairly well cleaned, but not hand-picked before the grains were stored in the silo^8^.

The Tel Tsaf silos as well as some other earlier storage facilities from the region (e.g., the Pre-Pottery Neolithic A granaries from Dhra' in Jordan^9,10^, and the evidence from the northern Levant^11^), also indicate that intensive human environmental intervention already existed during the Early Holocene, perhaps setting in motion processes that dramatically affected the region’s vegetal landscape^12^. Evidently, agricultural production was practiced at a scale capable of filling these storage facilities. Barley and wheat were the primary cultivars, but lentils and peas comprised important parts of the diet as well^8,13,14^. Undoubtedly, an operation of such a large scale would have needed a sophisticated system of production, possibly including fertilizers, irrigation systems, and field management practices, such as incorporating fallow periods into the crop rotations^8^. In turn, these features suggest a high degree of social stratification and commerce^1^. As will be demonstrated in this study, this was the social and economic milieu that made the development of orchard economy at Tel Tsaf possible.

In comparison with the extensive discussion of cereals^1,2,8^, and legumes^8,13^, very little is known about the arboreal vegetation at Tel Tsaf and its environs. The charred wood assemblage recovered by the first expedition during 1979, yielded only 21 specimens^15^ composed of (in descending order): *Quercus ithaburensis* (Mount Tabor oak), *Tamarix* spp. (tamarisk), *Populus euphratica* (Euphrates poplar), *Ziziphus lotus* (jujube), *Pistacia atlantica* (Atlantic pistachio), *Olea europaea* (olive), *Acacia albida* (white acacia) and *Pistacia lentiscus* (lentisk). A much larger assemblage comprised of hundreds of pieces of charred wood collected by the second expedition (2004–2007) is the focus of the current study.

According to the Principle of Least Effort (PLE) and models produced by Site Catchment Analyses (SCA), wood for everyday use (fuel, construction, toolmaking, etc.), like many other resources, was gathered locally with minimum effort^16–21^. By implication, wood found in an archaeological site is presumed to reflect its arboreal environment, both natural and cultivated. Only rarely was precious wood imported from afar and it was mainly used for the construction of prestige buildings or the manufacture of delicately crafted objects^22–24^.

Relying on these premises, we will draw on the large Tel Tsaf’s wood charcoal assemblage to reconstruct the natural arboreal vegetation near the site and demonstrate the practice of early fruit-tree horticulture in the Central Jordan Valley. It will be shown that Tel Tsaf provides one of the earliest examples of fruit tree cultivation worldwide.

## Tel Tsaf and its environs

### The site

**Figure 2:**
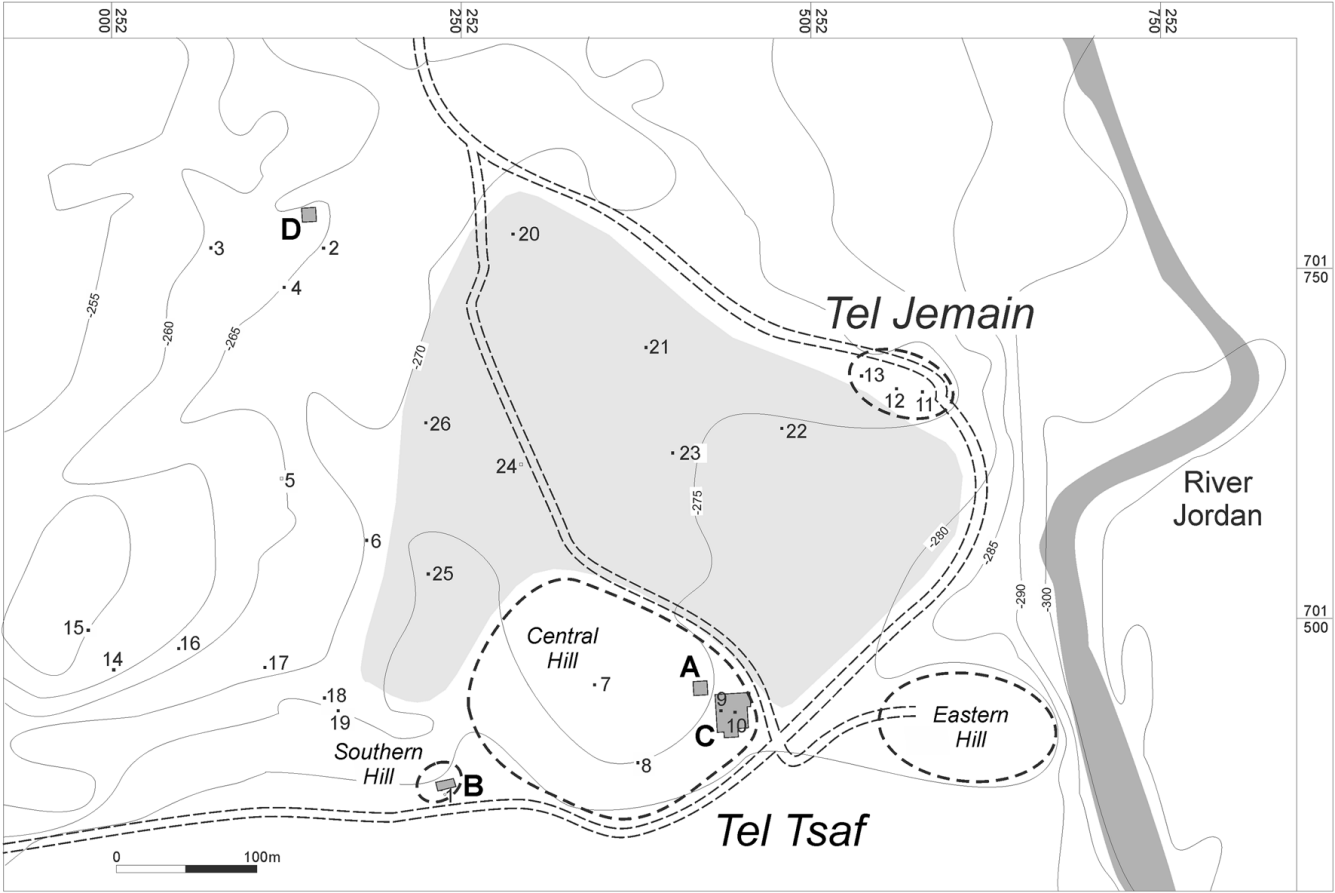
Map of Tel Tsaf and surroundings. Area C = settlement area, Area B = well. Gray area marks a recent cultivated area (Map by J. Rosenberg; taken from[^32^:Fig. 4.6]).

**Figure 3:**
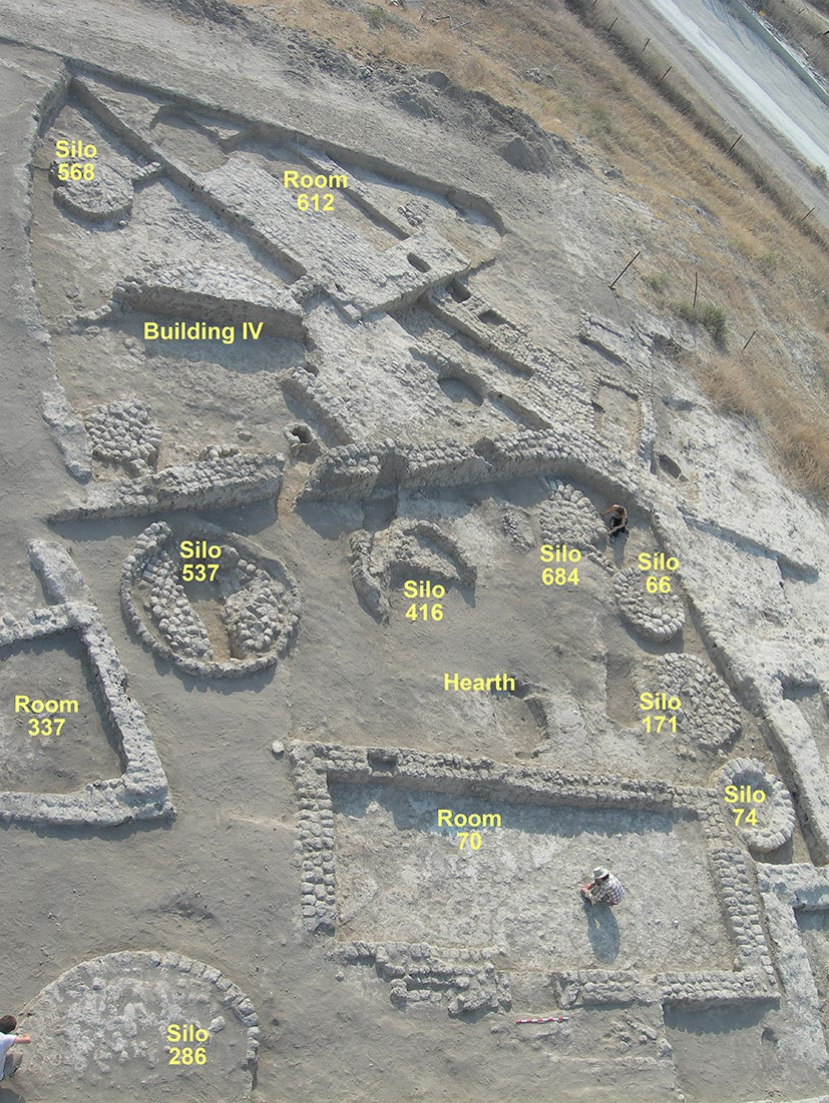
The village of Tel Tsaf; an overview of Area C with courtyard buildings, comprising rectangular and rounded rooms and rounded silos (Photo by Y. Garfinkel).

Tel Tsaf is located in the Central Jordan Valley, 32.5 km south of the Sea of Galilee (Fig. 1; Israel new grid 252391/701471), on the edge of the Jordan River’s west bank, and at an elevation of 270–280 m below mean sea level (Fig. 2). The site comprises three low hills and an expansive plain to the west and north, reaching about 20 hectares in total. Tel Tsaf had been investigated by three different expeditions: the first one was directed by Gophna, who excavated the site in 1979^26^. The second expedition, directed by Garfinkel, excavated at the site for four seasons, from 2004 to 2007^4,27^. In 2013 Rosenberg and Klimscha began to excavate the site^14,28^. The current report relates to charred wood remains from the second expedition that were recovered from two areas—B and C (the other two expeditions mainly focused on area C). Area B is located on the southern hill of the site (Fig. 2). Here, in a small sounding, the expedition unearthed a six m-deep well that reached the water table. Nearby, a living surface was found with concentrations of pottery and flint artifacts, but most of the sediments were eroded. Area C comprised an extensive, 750 m^2^ horizontal exposure. Here, the remains of several courtyard houses were found, consisting of a room or two, large rounded silos, and concentrations of hearths in the courtyard (Fig. 3). Another outstanding feature observed in Area C was the arrangement of rounded silos in rows, each with a storage capacity of several tons^1,2^.

Chalcolithic Tel Tsaf postdates the Wadi Rabah culture and predates the Ghassulian culture^29^, and is contemporaneous with Ubaid sites in northern Mesopotamia^30^. The chronology of the site indicates that occupation at Tel Tsaf began in the last quarter of the 8th millennium BP and ended in the first quarter of the 7th millennium BP (ca. 7200–6700 cal. BP^30,31^). The earliest occupation phases at the site were exposed in Area C, while Area B represents a slightly later occupation phase, yet the activity at both areas ceased around the same time^30^.

### Past and present environmental conditions

The site is located in the Irano-Turanian floristic region characterized by semi-arid steppe vegetation (i.e., Iranian steppes of the *Artemisia herbae-alba iranica*^33^). Some shrub elements belonging to the Mediterranean steppe-maquis zone, adjacent to the west, are present but sporadically. This steppic transitional zone receives between 200 and 400 mm annual precipitation (Fig. 1), which is relatively low but still sufficient for sustaining rain-fed agriculture. Coupled with its fertile soils and proximity to the Jordan River, the region is considered productive agricultural land (Fig. 2). Moreover, climate reconstructions for the Chalcolithic Mediterranean Levant region suggest slightly more humid conditions than today^34^. Either way, water, whether from precipitation, the Jordan River, or wells^4^ appears not to have been a constraint. Indeed, the site’s seed assemblages comprise economic taxa, such as barley (*Hordeum* sp*.*, *Hordeum vulgare*), wheat (*Triticum* sp., *Triticum dicoccum*, *Triticum monococcum*), lentil (*Lens culinaris*), and pea (*Pisum sativum*)^8,13,14^.

## Material and methods

### Anthracology (Charred wood remains)

Throughout the excavation, charcoal samples larger than 0.5 × 1 cm from secure stratigraphic contexts were collected, primarily by hand and dry sieving through a 2 mm mesh (which was systematically applied to all excavated sediments). Most of the samples were collected from Area C (Fig. 2), with only a few samples recovered from Area B. The charcoal samples’ taxonomic determination was accomplished on the basis of anatomical tissue structure studied at the most detailed taxonomic level. The specimens were cut and examined along three observational axes (transverse, tangential, and radial) using a stereoscopic Carl Zeiss SteREO Discovery.V20 microscope with magnifications of up to 360 × under oblique-angled top-illumination. A Scanning Electron Microscope (SEM: JOEL JSM-6300) was used when higher magnification was required. To obtain a representative assemblage, at least half of the specimens from every basket were analyzed. Each basket originated from a different archaeological context and contains at most ten specimens. Various sizes of samples were identified to prevent biases. Taxonomic determination was based on the identification of wood anatomical features (e.g., vessels and their arrangements, size and arrangement of rays, and the patterns of parenchyma and fibers) and their comparison with a south Levantine wood and charcoal reference collection (Steinhardt Museum of Natural History, Tel Aviv University). Wood anatomy atlases (e.g.,^35,36^) were also used to aid the identification process. Our study complies with relevant institutional, national, and international guidelines and legislation. The formal ethical requirement in Israel to analyze botanical material from an archaeological context is to get permission from the Israel Antiquity Authority (IAA), that is, a formal license. In this case, the Tel Tsaf excavation licenses are G-52/2004, G-31/2005, G-53/2006, and G-38/2007.

## Chronology

Though the site’s chronology is well defined by ceramic typology and ^14^C dates^30,31^, we wished to establish a more accurate chronological determination of the earliest phase of fruit-tree cultivation at the site. This attempt was partially motivated by suboptimal circumstances of eighteen dates presently available from the same contexts where the charcoal assemblage has been originated^30^. Two derive from olive pits, but they originated from Area B and thus represent a later occupation phase at the site. In Area C, where the earliest phases were exposed, only unidentified charcoals and cereal grains were dated^30^. Therefore, microscopic analysis was also geared to find young fruit-tree branches (twigs) in the Area C charcoal assemblage to serve as a short-lived organic specimen for ^14^C dating. The criteria for selection dictated that it would be a relatively small sample whose pith, xylem tissue, and bark are all observed. In this vein, a young common fig (*Ficus carica*) branch was identified and subjected to radiocarbon dating. The date was generated by Accelerator Mass Spectrometry (AMS) at the Beta Analytic Laboratory (Miami, Florida, no. Beta-585311). The radiocarbon age is reported in conventional radiocarbon years (before present = 1950), calibrated to calendar years (cal. BP^37,38^), and compared to previous dates obtained on charred wood fragments from Area C^30^ that were calibrated with the same program used in this study (OxCal 4.4, IntCal 20).

## Results

### The charcoal assemblages

A total of 622 charred wood samples were identified, 26 of which derive from the well in Area B and 596 from Area C. Altogether, 16 woody taxa were recorded (Table 1). Because Area B’s assemblage is significantly smaller than that of Area C’s, and since almost the same taxa were identified in both, we decided to focus on the larger assemblage of Area C.

**Table 1:** Identified charred material in absolute numbers and percentages at Tel Tsaf.

	Identified taxon	Area B		Area C	
		No. of specimens	%	No. of specimens	%
Native Mediterranean trees	*Quercus ithaburensis* (Mt. Tabor oak)	1	3.8	237	39.8
	*Quercus calliprinos* (evergreen kermes oak)			6	1.0
	*Quercus* spp. (oaks)			6	1.0
	*Crataegus* spp. (hawthorn)			9	1.5
	*Cercis siliquastrum* (Judas tree)	2	7.7		
	*Pistacia lentiscus* (mastic)			4	0.7
	*Pistacia* spp. (pistachio)			1	0.2
Semi-arid Irano-Turanian steppe taxa	*Tamarix* spp. (tamarisk)	4	15.4	133	22.3
	*Salsola* spp. (saltwort)	2	7.7	8	1.3
	*Suaeda* spp. (seepweed)	2	7.7	4	0.7
	*Acacia raddiana* (twisted acacia)			5	0.8
	*Capparis spinosa* (caper bush)			2	0.3
Riverbank trees	*Populus euphratica/Salix* (Euphrates poplar/willow)			59	9.9
	*Vitex agnus-castus* (chaste tree)			4	0.7
Fruit trees	*Ficus carica* (common fig)			51	8.6
	*Olea europaea* (olive)	12	46.2	38	6.4
	Unidentifiable	3	11.5	29	4.9
	Total	26	100	596	100

**Figure 4:**
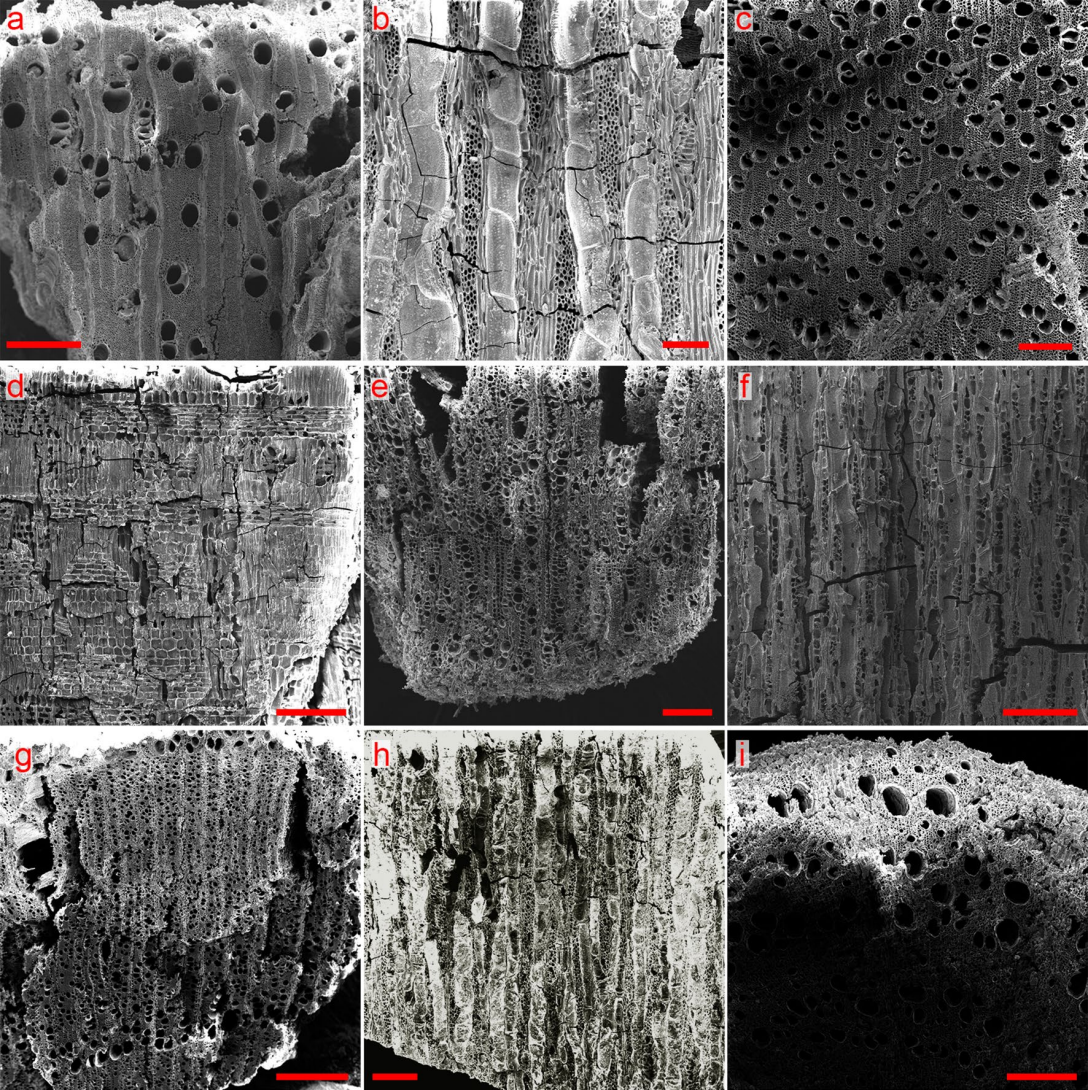
SEM images of charred-wood sections of taxa identified at Tel Tsaf, Area C. (**a**) *Ficus carica*, transverse, scale 500 μm. (**b**) *Ficus carica*, tangential, scale 100 μm. (**c**) *Salix/Populus*, transverse, scale 200 μm. (**d**) *Olea europaea*, radial, scale 200 μm. (**e**) *Olea europaea*, transverse, scale 200 μm. (**f**) *Olea europaea*, tangential, scale 200 μm. (**g**) *Cercis siliquastrum*, transverse, scale 500 μm. (**h**) *Cercis siliquastrum*, tangential, scale 200 μm. (**i**) *Quercus ithaburensis* (twig), transverse, scale 200 μm. Images were taken by M. Cavanagh using a Tescan VEGA3 LMH scanning electron microscope.

The most common taxon in this assemblage was *Quercus ithaburensis* (Mt. Tabor oak, 39.8%; Fig. 4), a native Mediterranean deciduous oak tree. The second most common taxon was *Tamarix* spp. which accounted for 22.3%. Although species from this genus can be found all over Israel, they thrive especially in wet habitats as well as in arid and saline environments. Tamarisk is a common tree on the banks of the Jordan River and the Sea of Galilee^39^. Other woody plant taxa identified in the assemblage common along the Jordan River are *Populus euphratica*/*Salix* (Euphrates poplar/willow, 8.6%; Fig. 4c) and *Vitex agnus-castus* (chaste tree, 0.7%). Other Mediterranean taxa occur in low frequencies and include *Crataegus* spp. (hawthorn, 1.5%), *Quercus calliprinos* (evergreen kermes oak, 1.0%), *Quercus* spp. (oak species, 1.0%), *Pistacia lentiscus* (mastic, 0.7%), and *Pistacia* spp. (pistachio species, 0.2%). Within the group of the semi-arid Irano-Turanian steppe vegetation, in addition to *Tamarix* spp., the following taxa were found: *Salsola* spp. (saltwort, 1.3%), *Acacia raddiana* (twisted acacia, 0.8%), *Suaeda* spp. (seepweed, 0.7%) and *Capparis spinosa* (bean caper, 0.3%). The assemblage also includes two important Mediterranean fruit trees: *Olea europaea* (olive, 6.4%; Fig. 4d–f) and *Ficus carica* (common fig, 6.5%; Fig. 4a,b).

### ^14^C dating

A young branch of common fig (i.e., a twig of *Ficus carica*) was subjected to AMS ^14^C measurement, yielding an age of 6060 ± 30 BP years (Table 2). Upon calibration, the age range in the 1σ interval is from 6960 to 6860 cal. BP^37,38^. A comparison with previous dates generated for charcoal specimens from Area C shows that the date obtained in this study is characterized by the narrowest age range (Table 2). It indicates that by identifying a twig in the charred wood assemblage, we may have managed to avoid the age of the tree and, therefore, achieved a more accurate date that could be considered as short-lived dating material.

**Table 2:** Radiocarbon dates of charred wood from Area C, Chalcolithic Tel Tsaf.

	Lab no	Date material	^14^C years (BP)	Calibrated age range (68.2% probability) BP	δ^13^C ‰	Reference
1	Beta-585311	Twig of *Ficus carica* (common fig)	6060 ± 30	6960–6860 cal. BP	− 27.6	This study
2	RT*-5477	Charred wood (taxon undetermined)	6085 ± 50	7150–6860 cal. BP	− 25.5	^30^
3	RT-5478	Charred wood (taxon undetermined)	6110 ± 75	7160–6890 cal. BP	− 20.0	^30^
4	RT-5479 (Rehovot)	Charred wood (taxon undetermined)	6150 ± 55	7160–6960 cal. BP	− 25.8	^30^

*RT = Weizmann Institute of Science in Rehovot, Israel.

## Discussion

### Reconstruction of the natural arboreal environment of Chalcolithic Central Jordan Valley

Charred wood remains recovered from archaeological excavations are often assumed to be remnants of fuel material since, regardless of their intended use (construction, preparing various wooden tools, etc.), most wood would have eventually been burned^40,41^ and preserved at the site as charcoal. Assuming that wood for everyday use was collected from the site’s vicinity^20,22^, our results indicate three types of habitats in the area: Mediterranean, semi-arid steppe, and riverbank.

The absolute dominance of Mediterranean trees, comprising more than 40% of Area C’s charcoal assemblage, indicates a well-developed Mediterranean woodland/maquis in Tel Tsaf’s catchment area. However, it is represented almost exclusively by the Mt. Tabor oak, a thermophilic deciduous oak typical of lower elevations of the south Levantine Mediterranean woodland. Interestingly, today, only a handful of specimens are observable in the environs of Tel Tsaf, underscoring a massive change in the region’s floral composition since the Chalcolithic period. Furthermore, its dominant position in the assemblage might indicate that it was originally procured for purposes of construction, firewood, and crafts. Other members typical to the southern Levant Mediterranean woodland/maquis identified in this study include different species of pistachio, evergreen oak, hawthorn and Judas tree.

Tamarisk, the second most common tree species in the assemblage, may have originated from riparian habitats, semi-arid habitats, or both. Typically, species of this genus were used for crafts, as observed for other Chalcolithic sites in the region (e.g.,^42^), and fuel^43^. Two other trees which probably grew along the Jordan River bank are Euphrates poplar and chaste tree. Within the seed assemblage, a few members of Cyperaceae (sedges) were identified^8^. Today, except for the riverbank proper, the region between the Sea of Galilee and the Dead Sea (i.e., the Central Jordan Valley) is characterized by semi-arid Irano-Turanian steppe vegetation and ~ 400 to 200 mm rainfall/year. Within the arid flora identified in this study, after tamarisk, the following taxa contributed to the assemblage: twisted acacia, bean caper, saltwort and seepweed.

### Evidence for horticulture ca. 7000 years cal. BP in the Central Jordan Valley

Five founder fruit trees established horticulture in the late prehistoric Levant^44–46^: olive (*Olea europea*), common fig (*Ficus carica*), grapevine (*Vitis vinifera*), date palm (*Phoenix dactylifera*), and pomegranate (*Punica granatum*). Of these, two—olive and fig—were found in the Tel Tsaf charred wood assemblage, offering a glimpse into the emergence of horticulture in the southern Levant.

#### Olive cultivation at Tel Tsaf

**Figure 5:**
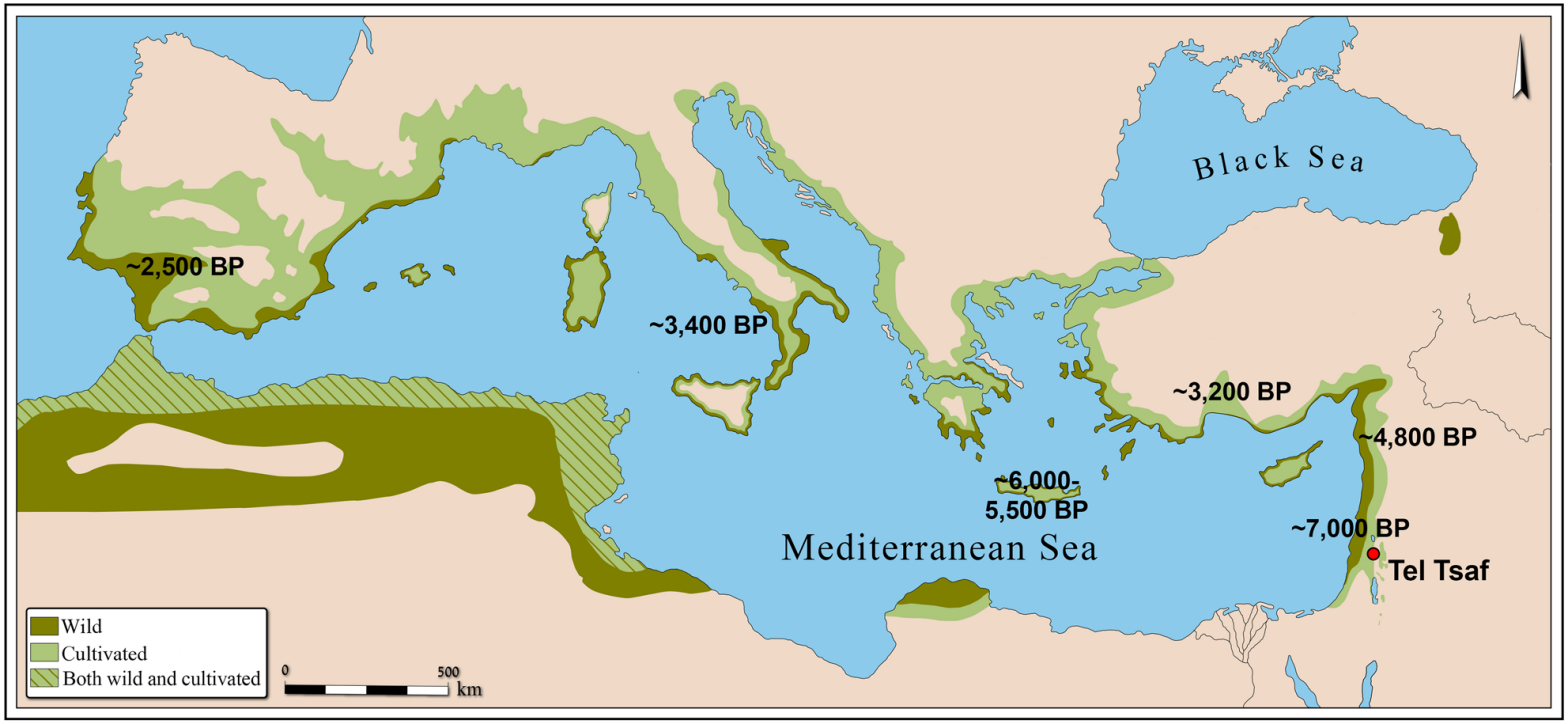
Geographical distribution of wild olive (*Olea europaea* subsp. *oleaster*) and cultivated olive in the Mediterranean Basin (modified after^48–50^) together with suggested dates for the beginning of olive horticulture in the Mediterranean regions^50^.

One of the most interesting finds in this study is the significant presence of charred olive wood remains. It is well-accepted, based on archaeobotanical research, that the existence of wood and/or its charcoal remains from fruit trees at a site point to their horticulture in its vicinity^22,23^^:103–104,^^47^. This is in contrast to seed, and fruit remains that do not necessarily indicate cultivation in the site environs, since they may derive from short or long-distance trade. Tellingly, the Central Jordan Valley is located outside the natural distribution area of wild olives (Fig. 5), and this appears to have been true in the 7th millennium BP as well. Consequently, the recovery of charred olive wood remains at Tel Tsaf provides strong evidence for olive orchards near the site. A few charcoal remains of olive as well as some olive stones were also reported in previous studies^8,13–15^.

It seems that Middle Chalcolithic Tel Tsaf provides the earliest charred olive wood remains in the Central and Lower Jordan Valley, followed by Late Chalcolithic sites such as Abu Hamid^51^ and Teleilat Ghassul^52^. Moreover, Chalcolithic olive oil production in the region is indicated also by the large amounts of olive-pressing waste in sites along the Jordan Valley^51^, as Pella^53^.

Looking beyond the Jordan Valley, the earliest archaeological and archaeobotanical evidence for olive oil production derives from the submerged site of Kfar Samir on the northern Israeli coast. The site was dated to the Late Pottery Neolithic/Early Chalcolithic period based on ceramic typology and 21 ^14^C dates (~ 7500 to 7000 BP^54,55^). Thousands of crushed olive pits, associated with stone basins and woven baskets identified as strainers^54^, were interpreted as large-scale olive oil extraction waste. The discovery of olive oil residues in a pottery vessel from the contemporary site of ‘Ain Zippori in the Lower Galilee^56^ provides further support for the production and consumption of olive oil in the northern parts of the country at this time. The recent identification of installations (still with thousands of olives inside them) which were used for the large-scale production of table olives at Hishuley Carmel (also a submerged site along the northern coast of Israel, Fig. 1) dated to ca. 6600 years cal. BP, may suggest that olive oil production evolved first and table olives followed^57^. Either way, unlike for the Jordan Valley, it remains indeterminate if these industries drew on wild or domesticated trees.

Numerous olive stones, olive wood remains, and crushing basins found at Chalcolithic sites in the Golan Heights^58^ and Samaria^59^, strongly suggest that olive horticulture was well established by ca. 6000 cal. BP in the southern Levant. This estimated date accords well with the seminal study conducted almost five decades ago by Zohary and Spiegel-Roy^44^, based mainly on the archaeobotanical evidence (charred seeds and wood) available at that time, which indicated that olive horticulture was already present in the type-site of Tuleilat el-Ghassul at no later than 6000 years BP.

A comprehensive palynological investigation covering the entire Mediterranean Basin showed that the southern Levant was a locus of primary olive cultivation as early as the first half of the 7th millennium BP^50^. The indication for this is a sudden increase in olive pollen, while other Mediterranean broadleaved trees (e.g., oaks and pistachios) remained more-or-less the same, thus rejecting a climate-related change^50^. The earliest of these anthropogenic olive pollen increases was registered at the Sea of Galilee, ca. 7000 cal. BP^60^, followed by other locations along the Jordan Valley rift—the Dead Sea^61^, the Hula Valley^62^, and Birkat Ram^60,63^—at ca. 6500 cal. BP. For the northern Levant, the suggested date of olive cultivation based on the palynological evidence is ca. 4800 cal. BP^50^. Genetic evidence, however, supports a slightly earlier date with a suggestion that the northern Levant was the locus of primary olive domestication^64^.

Thus, archaeological and botanical evidence suggests that olive cultivation began in northern Israel (Carmel coast and the Galilee) towards the end of the 8th millennium BP, during the Early Chalcolithic period, probably drawing on naturally occurring wild olive species [*Olea europaea* L. subsp. *europaea* var. *sylvestris* (Mill.) Lehr.]. A few centuries later, at the beginning of the Middle Chalcolithic period (ca. 7000 cal. BP), the settlers of Tel Tsaf engaged in full-fledged olive cultivation, indicated by their location outside *Olea europaea*’s natural distribution (Fig. 5). To accomplish this geographical shift, a transfer of both knowledge and genetic olive material from northern Israel to the Central Jordan Valley must have occurred.

The wild olive is considered a sensitive bioindicator for the Mediterranean bioclimatic zone^50,64–66^, usually found in hilly areas as part of the garrigue and woodland/maquis^33^. Cultivation expanded the species distribution (*Olea europaea* subsp. *Europaea* var. *sativa*) to higher and lower altitudes and latitudes, of which Tel Tsaf and the Jordan Valley is a particular instance^50^. Notwithstanding, olive requires at least 400 mm of annual rainfall to do well^67^. Given that the Chalcolithic period is presumed to have enjoyed a slightly more humid climate than today^34^, and due to the proximity of Tel Tsaf to the Jordan River (Fig. 2), it is possible that the olive orchards at site’s catchment area were either rain-fed, irrigated, or both. In any event, and as was already argued for other plants, it is clear that the period when a species spread beyond its natural wild distribution, can be taken as indicative of cultivation^11,68,69^.

#### Fig cultivation at Tel Tsaf?

Almost 10% of the charred wood assemblage of Tel Tsaf consisted of common fig (*Ficus carica*), complementing Gophna and Kislev’s earlier finds of common fig seeds (pips) at the site^13^. These archaeobotanical remains point to the consumption of figs at Tel Tsaf, but it is unclear whether they originate from local wild or cultivated trees or via trade. Three main reasons underlay this uncertainty: (1) *Ficus carica* occurs as both wild and feral riparian trees along the Jordan River in the vicinity of Tel Tsaf, (2) it is impossible to distinguish wild from domesticated fig on the grounds of wood anatomy and seed morphology^45,70^, and (3) the literature regarding on the timing of fig domestication produced inconclusive and sometimes contradictory results (more below).

Nevertheless, we propose that figs were cultivated at Tel Tsaf. This hypothesis is suggested by the significant occurrence of young branches that may have originated from pruning. Pruning is standard practice in fruit tree horticulture: it allows sunlight to reach all of a tree’s branches, keeps it at the desired size, and increases its fruit yield^67,71,72^. After pruning, the trimmed branches are removed to prevent the spreading of fungi and pests onto healthy trees, subsequently serving as a readily available fuel source, a practice still common among traditional Levantine societies^73,74^.

Lev-Yadun^70^ showed that unlike the tree’s seeds and fruit, common fig wood is a rare find in archaeological sites, comprising, at most, a small fraction of the wood assemblage. This scarcity of fig wood is attributed to its limited usefulness: it does not provide long and sturdy beams, and it is unknown to have been traded for other purposes. Therefore, when fig tree wood remains are found in an archaeological context, it may be deduced that fig trees grew nearby^70^. Early Bronze Age Tel Bet Yerah near the Sea of Galilee is a case in point (Fig. 1): a substantial number of young fig branches were found in the charcoal assemblage, suggesting fig cultivation was practiced^75^. Fig fruits can be eaten raw or in cooked form and can also be dried for later use. They are a source of several vitamins^10^ and as such would have provided a valuable nutritious complement to the diet of the Chalcolithic Tel Tsaf and Early Bronze Bet Yerah inhabitants.

Lev-Yadun^70^ also reviewed and discussed the previous hypotheses concerning the timing of common fig domestication: the classic and widely accepted hypothesis suggests that the package of five founder fruit trees that established Near-Eastern horticulture was domesticated during the Chalcolithic period, some 6000 years ago^11,44,46^. A dramatic hypothesis raised by Kislev et al.^76^ suggested that the common fig was domesticated in the lower Jordan Valley 11,400–11,200 years ago, already in the Pre-Pottery Neolithic A, i.e., even before the beginning of grain crop agriculture at about 10,500 years ago. This hypothesis was rejected by others^45,46,70,77–79^. Kislev et al. (2006) who found several seedless syconia, based their assumption on a botanical mistake, ignoring that all traditional common fig varieties produce in addition to early or late season seedless syconia lots of syconia with viable seeds when pollinated^71,78^, thus making it unacceptable. Furthermore, it has been shown that the inhabitants of another nearby site consumed wild figs during the Pre-Pottery Neolithic period^10^.

It is still unclear whether common fig domestication was an independent innovation that occurred at a specific time and place, gradually spreading throughout the Mediterranean Basin, or multiple culturally and genetically unrelated events. This indeterminacy is rooted in the ease of female common fig’s clonal propagation via branch cuttings and the lack of anatomical differences between wild and domesticated types, rendering modes of domestication and distinctions between primary and secondary domestication difficult to trace^70^. Since the Near East is generally regarded as a mono-cultural unit since the Pottery Neolithic^80^, it is unlikely to assume that the invention of horticulture could have occurred independently, several times, in this restricted area^79^.

### The archaeological meaning of fruit tree horticulture at Tel Tsaf

As illustrated by numerous case studies, the diversification of food and food habits is often linked with increased social complexity^9,69,81–84^. At Tel Tsaf, this goes hand in hand with numerous prestige objects that signal individuals’ and elites’ power, wealth, and status over other segments of the society^85–87^. At Tel Tsaf, two types of prestige items can be distinguished. The first type consists of imported objects procured through long-distance exchange networks, including obsidian, rare-mineral beads, Ubaid pottery, and a copper awl. Prestige objects of this type derive their value from being rare and exotic. The second type of prestige objects consists of local but highly-invested products, like elaborately decorated pottery and fruits. The prestige attached to these objects derives from the extra time and effort that went into their production.

Ultimately, all prestige goods and products can be ostentatiously exhibited and consumed at feasts, which according to the animal bone finds, seem to have been extensively practiced at Tel Tsaf^3^. Such events may have included meat, fruit, and beverages served in elaborate pottery dishes, while the participants may have adorned exotic beads. In this way, the wealthy elite at Tel Tsaf manifested and displayed its superiority, underscoring the higher quality of their nutrition and their distinguishing material and aesthetic attributes. The display and consumption of fruits and fruit products such as olive oil are likely to have been integral to these shows of extravagance. It is worth recalling Hayden’s^88^ suggestion that feasts provided considerable momentum to early domestication.

When integrating fruit trees into an agro-economic system that relies mostly on grain crops as was the case for Tel Tsaf, it is important to consider a few key themes, some of which relate to the differences between annual grain crops agriculture and fruit tree horticulture. Fruit tree cultivation is a long-term investment with a relatively delayed return^69^. It is possible that a fruit tree plantation would not assume its full yield potential within the short adult lifetime of the planter 7000-years-ago, due to the long juvenile period of some of the fruit tree types, even if clonally propagated^79^. In addition, fruit trees cannot be rotated like annual plants between different plots, and therefore special care must be taken when allocating the land for a fruit tree plantation^79^. Furthermore, unlike annual grain crops, orchards are long-lived and, therefore, have considerable implications for land ownership and heritage systems. All these features are calling for more elaborate social contracts and institutions^69^. The discovery at Tel Tsaf of a seal (found at building II’s courtyard), 140 clay sealings and a clay sealing stamped with two different seals that are not known from any other site of this period in the southern Levant, is therefore not surprising^5,6^. All of these findings, along with the unprecedented phenomenon of large silos^1^, indicate a rise in social and economic complexity, as was already shown for other storage facilities in earlier cases from the region^9^. In this respect, the cultivated trees and their products can be placed alongside craft specialization^69^. In the Chalcolithic Jordan Valley, this probably included metallurgy^7^, textiles^42^, and the importation of exotic products^4,89^.

To support larger populations and generate more wealth, people had to grow more food. This can be accomplished via several strategies: by the expansion of the amount of land under cultivation; by the extraction of more agricultural products from a given unit of cultivated land (e.g.,^8^); and by growing crops with long shelf lives^90^. Tel Tsaf is a case in point: its cultivated olive and fig trees produced products with long shelf lives, like table olives, olive oil, and dried figs and, therefore, are highly suitable for long-distance trade and taxation^91^, leading eventually to the accumulation of wealth and a more complex social-economical organization^90,91^.

## Summary and conclusions

Today, the olive is considered the most prominent and probably the economically most important fruit tree of the Mediterranean Basin. Cultivation caused the species’ (*Olea europaea*) distribution to expand into areas otherwise beyond its natural habitats (Fig. 5). The charcoal assemblage of Tel Tsaf provides the earliest evidence of olive cultivation outside its natural distribution. It also offers evidence for early cultivation of common fig (*Ficus carica*), both dated to 7000 cal. BP. Tel Tsaf’s rich material culture and unparalleled prosperity constitute the background against which early fruit tree horticulture crystallized, which seems to have had several important implications:

1. On the practical level, the inhabitants of Tel Tsaf enjoyed better nutrition, as the traditional cereals and legumes diet was augmented by fruits, which are high in nutrients (such as vitamins and fibers). Moreover, olive oil’s suitability for storage, coupled with the possibility of curing olives and drying figs, may have guaranteed a long-term and stable supply of these products. Of course, one could acquire these resources by foraging wild fruits, but yields would have been limited, geographically restricted, and of lower quality.
2. Unlike fields of annual plants (e.g., cereals and legumes), orchards constitute enduring features in the landscape and call for high initial investments. These features inevitably complicate matters of land ownership and inheritance, calling for more elaborate social contracts and institutions. A degree of local administrative practices at Tel Tsaf was already evident in the large storage facilities and the presence of a stamp seal. Historically, olive oil, table olives, and dry figs were highly suitable for long-distance trade and taxation, which led to the accumulation of wealth and possibly a rise in social-economic complexity.

## Data Availability

All data generated or analyzed during this study are included in this article (Tables 1, 2).
